# Correlates of Adverse Childhood Experiences Among Children in Non-Hispanic White, Non-Hispanic Black and Hispanic Immigrant Households in the United States

**DOI:** 10.1007/s10903-025-01785-9

**Published:** 2025-10-28

**Authors:** Angela G. Campbell, Wura Jacobs

**Affiliations:** Indiana University Bloomington, Bloomington, USA

**Keywords:** Adverse childhood experience, ACEs, Immigrant, Foreign-born, Minority health, Health disparities, Immigrant health, Structural determinants

## Abstract

As the immigrant population in the United States increases and diversifies, the necessity of examining adverse childhood experiences (ACEs) by racial and ethnic group within the immigrant population has become apparent. This study examines patterns and structural correlates of ACEs among children in Hispanic, Non-Hispanic (NH) Black, and NH White immigrant households. The National Survey of Children’s Health (2016–2023) was utilized to obtain a nationally representative sample of children (ages 0–17) for a cross-sectional study (*N* = 32,094). Descriptive statistics and multinomial logistic regression models were used to examine the correlates of low ACEs (1 ACE) and high ACEs (≥ 2 ACEs). NH Black immigrant households had the highest percentage of children experiencing high (12.7%) and low ACEs (28.2%). Each incremental increase in income was associated with reduced odds of high ACEs exposure among NH Black immigrant children, but NH White immigrant children only benefited from increased income if they were in the highest income bracket (< 400% of the poverty line). English as the primary household language was associated with increased odds of high ACEs exposure among all immigrant groups. This study documents patterns and structural correlates of ACEs among children in Hispanic, NH Black, and NH White immigrant households. ACEs prevalence patterns mirror those in the general population, with children in NH Black immigrant households at highest risk. The impact of sociodemographic and structural factors varied across immigrant racial and ethnic groups, pointing to the complex interplay between socio-structural factors and immigrant group-specific stressors.

## Introduction

Adverse Childhood Experiences (ACEs) are not determined by a child’s race, ethnicity, class, or gender; however, racial and ethnic differences exist in the prevalence of ACEs [1–4], levels, and types of adversities [3–5]. A key driver of racial and ethnic disparities in prevalence of ACEs are the social and structural determinants of health, which disproportionately impact racial and ethnic minoritized population subgroups [1, 5, 6]. The social and structural determinants of health, including socioeconomic status, neighborhood environment, food and housing, healthcare access, and systemic racism, play a critical role in the incidence of ACEs [6, 7].

Children living in immigrant households in the U.S. may be vulnerable to ACEs due to a variety of stressors unique to their family migration history, motivation (e.g., education and economic pursuits, asylum seeking, fleeing hardship), and experience [1, 8–10]. Particularly for immigrant children from racial and ethnic minoritized backgrounds, migration-related stressors (e.g., economic hardship, language barriers, socio-political context) in addition to systemic issues such as discrimination and persisting racial and ethnic inequities in resource accessibility in the U.S. can exacerbate their risk for ACEs [8, 10] and poor health overall [1].

Despite ample evidence connecting race and ethnicity, socioeconomic context, and structural determinants of health to ACEs [1, 11, 12], and evidence showing that immigrant and native U.S. families differ on structural and socioeconomic indicators [13, 14], very few studies have examined the correlates of ACEs specifically among children from immigrant families in the U.S. Slopen et al., [1] examined racial and ethnic differences in ACEs, comparing correlates of ACEs among children of U.S.-born and immigrant parents relative to their non-Hispanic White counterparts using data from The 2011–2012 National Survey of Children’s Health (NSCH) [1]. This important study highlighted the pivotal intersectional impact of income and education on ACEs exposure, showing the effect varied based on immigrant status.

This current study extends the work of Slopen and colleagues in three crucial ways. First, we examine the structural and sociodemographic correlates of ACEs specifically among children in immigrant households by race and ethnicity. Second, in a departure from the normative and increasingly discouraged standard of comparing minoritized racial and ethnic groups to a dominant racial group (e.g [15]).,, we employed a stratified approach for exploring the unique correlates of ACEs to better understand the structural and social inequities at play within groups [16–18]. Third, we employ recent (2016–2023) and more robust data to reflect current sociodemographic, policy, and economic contexts of immigrants in the U.S.

By identifying the determinants specific to each racial and ethnic group, this study aims to contribute to a more nuanced understanding of the structural contexts and characteristics associated with ACEs among racially and ethnically diverse immigrant households. Given that perceptions surrounding ACEs are cultural, contextual, and linked to the social and structural determinants of health [19], we hypothesize that there will be commonalities and unique determinants of ACEs among children in Hispanic, non-Hispanic (NH) Black, and NH White immigrant households.

## Methods

### Study Population

This study utilized data from the National Survey of Children’s Health (NSCH), a nationally representative survey of children in the U.S. aged 0–17 who are not institutionalized [20]. The survey was designed by the Health Resources and Services Administration’s Maternal and Child Health Bureau and is administered annually via internet and mail by the U.S. Census Bureau [21]. The survey is administered in both English and Spanish and can be completed online or via mail [22]. The 2011–2012 version of the NSCH offered additional languages, but only 0.2% of respondents utilized this service, so it was discontinued [23]. Although the study samples children, the answers to all questions were collected from an adult representative within the child’s household [22]. Additional information regarding the NSCH survey design and methodology can be found elsewhere [22].

The NSCH had 50,212 respondents in 2016 (response rate of 40.7%) [24] and 55,162 respondents in 2023 (response rate 35.8%) [25]. The 2017–2022 surveys had a range of 21,599 observations in 2017 to 54,103 observations in 2022. The NSCH data for 2017 had fewer observations, but it still had a response rate of 43.1%, which is comparable to other years [26]. The survey data is collected online and via mail so it is not likely that response rates were impacted by Covid [22]. The majority of these households did not contain a foreign-born parent. Therefore, to ensure adequate sample size, we pooled survey data from 2016 to 2023. As in previous studies using the NSCH data, we defined children in immigrant households as those with at least one foreign-born biological or adopted parent [1, 27]. We do not include measures of the status of both parents to allow for inclusion of single parent households. Additionally, we do not control for family structure as divorce is one of the ACEs measures and this is highly colinear with family structure. The analytic dataset was restricted to children who were identified as NH White, NH Black or Hispanic. Our initial sample restriction left a sample of 35,373 observations. Next, all observations with missing data on one of the variables included in the final models were dropped. This step led to 3,279 observations being dropped, which was 9.3% of the sample (descriptives in Appendix A). The final sample included 32,094 children living in households with one foreign-born biological or adopted parent. Within this sample, 11% (*N* = 3,514) of the children were also foreign-born.

### Measures

The dependent variable for this analysis was ACEs exposure. The NSCH has nine ACEs questions that are present in all survey years pooled. The NSCH measured all ACEs items dichotomously with the exception of food/housing insecurity where a response of “somewhat often/very often” was recoded as positive ACEs exposure [28, 29]. In alignment with other studies utilizing the NSCH data [30], we generated a categorical variable of total ACEs exposure with the following categories: 0 or no ACEs exposure, 1 ACE exposure, ≥ 2 ACEs exposure. This categorization was chosen so that we are in alignment with previous literature utilizing the NSCH data [11, 30] and because the number of children with greater than 2 ACES was not large enough to split by race/ethnicity (Appendix C).

Other study covariates included child age (0–5, 6–11, 12–17), child biological sex, child health (1 = excellent/very good/good, 0 = fair/poor), child insurance type (public only, private only, public and private, and uninsured.), highest parent education (< 12 years, high school graduate, some college or more), primary household language (English vs. Spanish/Other), poverty level (< 100% federal poverty line (FPL) (“poor”), 100%−199% FPL (“near poor”), 200%−399% FPL (“middle income”), 400%+ FPL (“high income”)), and past year household receipt of public benefits (0 = no benefits received, 1 = household received benefits from Women Infants and Children (WIC)/Temporary Assistance for Needy Families (TANF)/Supplemental Nutrition Assistance Program (SNAP)). The descriptives table also includes variables for the sample child’s nativity (“US-born,” “Foreign-born,” and “missing,”) and household nativity status, (“two foreign-born parents,” “one foreign born parent and one US-born parent,” “non-traditional family structure with at least one foreign-born parent,” and “two parents, but one has a missing nativity status.”

### Statistical Analysis

Descriptives statistics and multinomial logistic models were utilized for analysis. The NSCH administrators imputed race and ethnicity using hot deck imputation method and imputed federal poverty level using a sequential regression imputation method [31]. Hot deck imputation is a statistical technique whereby missing data is filled in using information from a unit that is observed to be similar in other dimensions [32]. This method is also utilized by the U.S. Census bureau for other datasets such as the Current Population Survey and National Health and Nutrition Examination Survey [32]. To account for the multiple imputations of the poverty variable, *weighted mi* commands were executed in STATA for the weighted percentages of federal poverty in the descriptives table as well as the multinomial regressions that utilized federal poverty level as a covariate. Multinomial regression models were utilized for analysis rather than ordinal regression models because at least one of the stratified models violated the proportional odds assumption. All models control for year of survey, child health, child age, child sex, child insurance type, highest parent education, primary household language, poverty level and past year household receipt of public benefits. In this study, we operationalized race and ethnicity as a categorical variable to identify population groups with systemically different resource accessibility and socioeconomic context [33]. Hence, instead of using interactions terms that require designating a normatively advantaged group as reference (e.g., non-Hispanic White), we conducted separate regression models for each racial and ethnic group [17, 18]. Multicollinearity was not detected as assessed with VIF < 5 [34]. All analyses were executed in Stata 18 and weighted to be nationally representative. This study was approved by the university Institutional Review Board as not human subjects research (IRB Protocol #23134).

### Results

The majority of immigrant households reported their child was in very good or excellent health; 93.3% for NH White, 89.7% for NH Black and 84.9% for Hispanic (Table 1). A larger proportion of Hispanic households reported highest parental education as high school education or less (55.1%) compared to NH White and NH Black households (12.1% and 22.3%, respectively). NH White and NH Black households had similar proportions of respondents whose primary household language was not English (22.9% and 21.1%, respectively), while 67.0% of Hispanic households spoke a language other than English. Both Hispanic and NH Black households had a higher percentage of children who did not have some form of private insurance (62.5% and 45.6%, respectively) compared to NH White households (26.2%). A similar proportion of Hispanic and NH Black households received public benefits (39.0% and 36.8%, respectively), while only 14.6% of NH White households receive public benefits. More than half of Hispanic and NH Black households (64.6% and 53.1% respectively) were categorized as low income or nearly poor (living below 200% of the poverty line), while only 26.9% of NH White households were in this income bracket.

**Table 1:** Study participant characteristics from The 2016–2023 NSCH survey by immigrant race and ethnicity*

Variables	Immigrant Non-Hispanic White (*N* = 13,951) % [CI]	Immigrant Non-Hispanic Black (*N* = 3,518) % (CI)	Immigrant Hispanic (*N* = 14,625) % (CI)		Total Sample (*N* = 32,094) % (CI)
*Sample Child Age*				***	
0–5 years	33.0 [0.31,0.35]	34.1 [0.31, 0.37]	27.0 [0.26, 0.29]		29.4 [0.28, 0.31]
6–11 years	34.8 [0.33, 0.36]	31.7 [0.29, 0.35]	34.8 [0.33, 0.37]		34.4 [0.33, 0.36]
12–17 years	32.2 [0.31, 0.34]	34.2 [0.31, 0.37]	38.1 [0.36, 0.40]		36.2 [0.35, 0.37]
*Sample Child Biological Sex*				NS	
Female	49.0 [0.47, 0.51]	47.0 [0.44, 0.50]	49.0 [0.47, 0.51]		48.7 [0.48, 0.50]
Male	51.0 [0.49, 0.53]	53.1 [0.50, 0.56	51.0 [0.49, 0.53]		51.3 [0.50, 0.53]
*Sample Child Nativity*					
US-Born	86.5 [0.85, 0.88]	82.4 [0.80, 0.85]	88.9 [0.88, 0.90]		87.4 [0.87, 0.88]
Foreign-Born	13.0 [0.12, 0.14]	17.4 [0.15, 0.20]	10.7 [0.10, 0.12]		12.2 [0.11, 0.13]
Missing	0.5 [0.002, 0.01]	0.3 [0.001, 0.01]	0.4 [0.002, 0.01]		0.4 [0.003, 0.01]
*Household Parent Nativity*				***	
Two foreign-born parents	37.2 [0.36, 0.39]	54.9 [0.52, 0.58]	50.5 [0.49, 0.52]		48.1 [0.47, 0.50]
One foreign-born and one US-born parent	50.4 [0.49, 0.52]	15.5 [0.14, 0.18]	25.1 [0.24, 0.27]		29.5 [0.28, 0.31]
Non-traditional family structure with at least one foreign-born parent	12.2 [0.11, 0.13]	28.9 [0.26, 0.32]	23.9 [0.23, 0.25]		22.0 [0.21, 0.23]
Two parents but one has a missing nativity status	0.2 [0.001, 0.003]	0.7 [0.004, 0.01]	0.5 [0.003, 0.01]		0.5 [0.003, 0.01]
*Child is in Excellent or Very Good Health*				***	
Yes	93.3 [0.92, 0.94]	89.7 [0.88, 0.91]	84.9 [0.84, 0.86]		87.5 [0.87, 0.88]
No	6.7 [0.06, 0.08]	10.3 [0.09, 0.12]	15.1 [0.14, 0.17]		12.5 [0.12, 0.13]
*Child Insurance Type*				***	
Public only	21.6 [0.20, 0.23]	38.3 [0.35, 0.41]	50.3 [0.49, 0.52]		42.0 [0.41, 0.43]
Private only	70.5 [0.69, 0.72]	49.0 [0.46, 0.52]	32.9 [0.31, 0.34]		43.9 [0.43, 0.45]
Public and private	3.3 [0.03, 0.04]	5.4 [0.04, 0.07]	4.6 [0.04, 0.05]		4.4 [0.04, 0.05]
Uninsured	4.6 [0.04, 0.05]	7.3 [0.06, 0.09]	12.2 [0.11, 0.14]		9.7 [0.09, 0.11]
*Household highest level of education*				***	
< 12 years	4.1 [0.03, 0.05]	7.1 [0.05, 0.09]	34.6 [0.33, 0.36]		23.6 [0.22, 0.25]
High school graduate	8.0 [0.07, 0.09]	15.2 [0.13, 0.18]	20.5 [0.19, 0.22]		16.8 [0.16, 0.18]
Some College or More	88.0 [0.87 0.89]	77.7 [0.75, 0.80]	44.9 [0.43, 0.47]		59.6 [0.58, 0.61]
*Household Language*				***	
English	77.1 [0.76, 0.79]	78.9 [0.76, 0.81]	33.0 [0.31, 0.35]		49.8 [0.49, 0.51]
Other	22.9 [0.21, 0.24]	21.1 [0.19, 0.24]	67.0 [0.65, 0.69]		50.2 [0.49, 0.51]
*Household has received public benefits in the last year*				***	
Yes	14.6 [0.13, 0.16]	36.8 [0.34, 0.40]	39.0 [0.37, 0.41]		33.1 [0.32, 0.34]
No	85.4 [0.84, 0.87]	63.3 [0.60, 0.66]	61.0 [0.59, 0.63]		66.8 [0.66, 0.68]
*Federal poverty line* ^a^				***	
Low income	11.3 [0.10, 0.13]	25.0 [0.22, 0.28]	32.9 [0.31, 0.35]		26.8 [0.25, 0.28]
Nearly poor	15.6 [0.14, 0.17]	28.1 [0.25, 0.31]	31.7 [0.30, 0.34]		27.5 [0.26, 0.29]
Middle income	26.3 [0.25, 0.28]	25.2 [0.23, 0.28]	22.5 [0.21, 0.24]		23.8 [0.23, 0.25]
High income	46.8 [0.45, 0.49]	21.6 [0.19, 0.24]	12.9 [0.12, 0.14]		21.9 [0.21, 0.23]

*Table shows weighted percentages.

^a^Weighted percentage calculated using mi commands in STATA to account for multiple imputation.

*p<.05, **p<.01, ***p<.001.

NS = Not Significant.

**Fig. 1:**
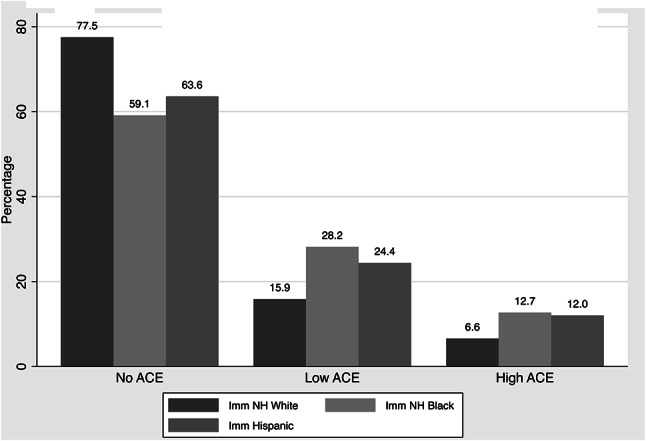
Prevalence of ACE category by race/ethnicity and immigration status

The prevalence of exposure to high ACEs (defined as ≥ 2 ACEs) was greatest among NH Black children (12.7%), followed by Hispanic children (12.0%) and then NH White children (6.6%) (Fig. 1). The same pattern was seen for low ACEs exposure (defined as having 1 ACE), with 28.2% of NH Black children having low ACEs exposure, followed by 24.4% of Hispanic children and 15.9% of While children. These differences were significantly different using a chi-square test (*p* <.001).

**Fig. 2:**
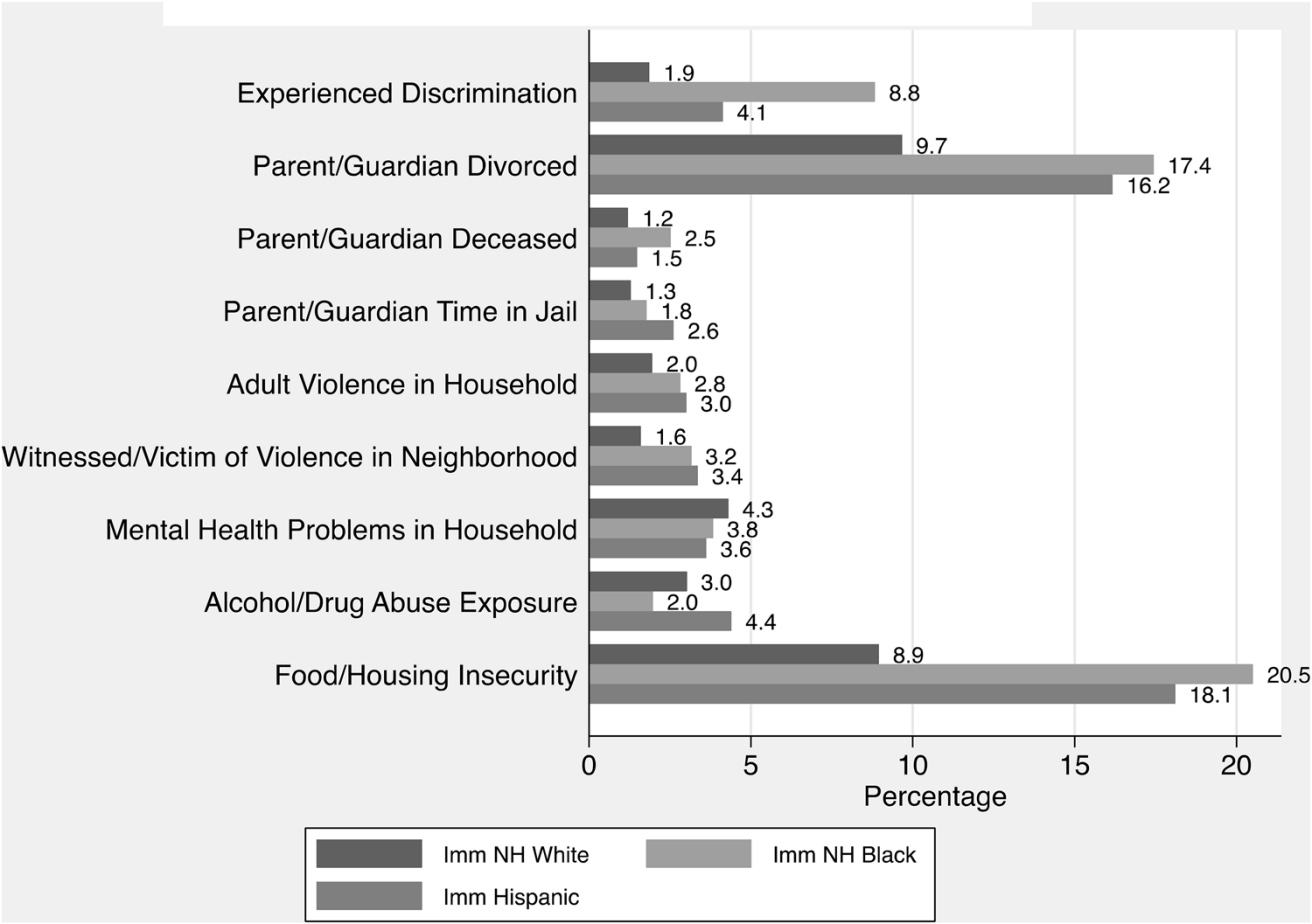
ACES by race/ethnicity and immigration status

Parent/guardian divorce was the most reported ACEs type for NH White children, but food/housing insecurity was the most common ACEs type for Hispanic (18.1%) and NH Black (20.5%) children living in immigrant households (Fig. 2). After food/housing insecurity, the next three most common ACEs types among NH Black children were divorce (17.4%), discrimination (8.8%), and mental health problems in the household (3.8%). Among Hispanic children, divorce (16.2%), alcohol or drug abuse exposure (4.4%) and discrimination (4.1%) were most reported. Among NH White children, after parental divorce, the top three ACEs reported were food/housing insecurity (8.9%), mental health problems in the household (4.3%) and alcohol or drug abuse exposure (3.0%).

Across all groups, children in very good or excellent health had reduced odds of high ACEs (Table 2). English as the primary household language was associated with increased odds of high ACEs for Hispanic and Black children (OR = 1.98; 95%CI (1.56, 2.50) and OR = 2.01; 95%CI, (1.67, 3.31) respectively). For NH White children, English as the primary language was associated with increased odds of both high and low ACEs (OR = 2.35; 95%CI (1.67, 3.31) and OR = 1.36; 95%CI (1.05, 1.78) respectively).

**Table 2:** Multinomial models comparing low (L) or high (H) ACE to no ACE by Racial/Ethnic Group*

Variables Sample Child Age		Immigrant Non-Hispanic White (*N* = 13,951)		Immigrant Non-Hispanic Black (*N* = 3,518)		Immigrant Hispanic (*N* = 14,625)	
		OR	95% CI	OR	95% CI	OR	95% CI
0–5 years		ref		ref		ref	
6–11 years	L	1.50**	(1.17, 1.92)	1.27	(0.89, 1.81)	1.38**	(1.10, 1.73)
	H	3.20***	(2.12, 4.82)	1.46	(0.84, 2.54)	2.36***	(1.70, 3.26)
12–17 years	L	2.51***	(2.0, 3.17)	2.04***	(1.45, 2.88)	1.91***	(1.53, 2.39)
	H	9.15***	(6.24, 13.4)	4.17***	(2.47, 7.04)	5.04***	(3.69, 6.87)
*Sample Child Biological Sex*							
Female		ref		ref		ref	
Male	L	0.87	(0.72, 1.05)	0.99	(0.75, 1.30)	0.89	(0.75, 1.06)
	H	1.21	(0.93, 1.57)	0.94	(0.64, 1.39)	0.82	(0.67, 1.02)
*Child is in Excellent or Very Good Health*							
Yes	L	0.56**	(0.38, 0.83)	0.64	(0.39, 1.03)	0.50***	(0.39, 0.64)
	H	0.52**	(0.34, 0.81)	0.25***	(0.14, 0.45)	0.42***	(0.31, 0.56)
No		ref		ref		ref	
*Household highest level of education*							
< 12 years		ref		ref		ref	
High school graduate	L	0.92	(0.44, 1.94)	0.64	(0.30, 1.38)	1.05	(0.82, 1.34)
	H	0.69	(0.33, 1.43)	0.69	(0.26, 1.79)	0.93	(0.70, 1.25)
Some College or more	L	0.95	(0.48, 1.90)	0.46*	(0.22, 0.96)	1.03	(0.82, 1.30)
	H	0.58	(0.31, 1.10)	0.57	(0.24, 1.35)	1.03	(0.78, 1.36)
*Primary Household Language*							
English	L	1.36*	(1.05, 1.78)	1.17	(0.81, 1.69)	1.14	(0.93, 1.41)
	H	2.35***	(1.67, 3.31)	2.01*	(1.12, 3.61)	1.98***	(1.56, 2.50)
Spanish/Other		ref		ref		ref	
*Child Insurance Type*							
Private only		ref		ref		ref	
Public only	L	1.35	(0.99, 1.85)	1.33	(0.90, 1.94)	1.18	(0.93, 1.49)
	H	2.10***	(1.46, 3.02)	1.80*	(1.08, 3.01)	1.2	(0.87, 1.66)
Public and private	L	1.93	(1.17, 3.19)	0.76	(0.39, 1.46)	1.59*	(1.08, 2.34)
	H	2.56**	(1.37, 4.78)	0.94	(0.35, 2.58)	1.72*	(1.07, 2.77)
Uninsured	L	1.18	(0.73, 1.93)	1.12	(0.66, 1.90)	1.33	(0.96, 1.82)
	H	1.06	(0.56, 2.00)	0.98	(0.43, 2.23)	1.63*	(1.09, 2.43)
*Household has received public benefits in the last year*							
Yes	L	1.31	(0.92, 1.86)	1.15	(0.80, 1.64)	1.78***	(1.44, 2.20)
	H	2.13***	(1.43, 3.18)	1.22	(0.77, 1.92)	2.37***	(1.85, 3.05)
No		ref		ref		ref	
*Federal poverty line*							
Low income		ref		ref		ref	
Nearly poor	L	0.77	(0.53, 1.13)	1.03	(0.64, 1.65)	0.95	(0.76, 1.19)
	H	0.84	(0.52, 1.36)	0.56*	(0.32, 0.96)	0.93	(0.71, 1.22)
Middle income	L	0.79	(0.52, 1.18)	1.04	(0.62, 1.75)	0.72*	(0.54, 0.96)
	H	0.97	(0.53, 1.76)	0.55*	(0.32, 0.95)	0.76	(0.55, 1.06)
High income	L	0.49***	(0.33, 0.72)	0.84	(0.48, 1.49)	0.60*	(0.40, 0.89)
	H	0.38**	(0.21, 0.68)	0.39**	(0.21, 0.74)	0.35***	(0.21, 0.56)

*p <.05, **p <.01, ***p <.001.

Models were also adjusted for year of survey. Analyses are weighted to be nationally representative.

Being uninsured was associated with increased odds of high ACEs among Hispanic children [OR = 1.63 95%CI (1.85, 2.43)]. Among NH White Children, having both “public only” and a “mix of public and private” insurance was associated with increased odds of high ACEs [OR = 2.10 95%CI (1.46, 3.02) and OR = 2.56, 95%CI (1.37, 4.78)], respectively). Having “public only” insurance was associated with increased odds of high ACEs for NH Black children [OR = 1.80; 95% CI (1.08, 3.01)]. Receipt of public benefits was associated with increased odds of both high [OR = 2.37 95%CI (1.85, 3.05)] and low ACEs [OR = 1.78 95%CI (1.85, 3.05)] for Hispanic children. For NH White children, receipt of public benefits was associated with increased odds of high ACEs [OR = 2.13, 95%CI (1.43, 3.18)].

Among NH Black children, each successive increase from low to high income was associated with significantly reduced odds of high ACEs (Table 2). For Hispanic children, being in a middle or high-income household was associated with a significantly reduced odds of low and high ACEs. For NH White children, only those in high-income households (≥ 400% of the poverty level) had significantly reduced odds of low [OR = 0.49; 95%CI (0.33, 0.72)] and high [OR = 0.38; 95% (CI, 0.21, 0.68)] ACEs; a 51% and 62% reduction respectively.

## Discussion

This study examined the structural and sociodemographic correlates of 0/no adverse childhood experiences (ACEs), 1 ACE (low), and ≥2 ACEs (high) specifically focusing on NH White, NH Black, and Hispanic immigrant households [35]. Similar to patterns reported in previous work [1], NH Black immigrant children exhibited the highest prevalence of high ACEs (i.e., experience of two or more childhood adversities), followed by Hispanic and NH White immigrant children. This pattern mirrors what is reported among U.S. born populations where greater childhood adversity (i.e., high ACEs) is reported among minoritized racial and ethnic groups [1, 4]. While Slopen et. al. [1] identified household income as the major correlate for high ACEs, our within-group examination highlighted important similarities and differences in both low and high ACEs corelates among the different racial and ethnic immigrant groups that would have been unobserved in interaction models.

Higher income was associated with lower risk of ACEs for all immigrant groups; however, the protective effect was not consistent across income levels. Among children in NH White households, only those in the highest income bracket experienced significantly reduced risk of low and high ACEs exposure. In contrast, children in NH Black immigrant households showed a more graded pattern, where each successive increase in income level was associated with a lower risk of high ACEs exposure. For children in Hispanic immigrant households, the protective effect of income on ACEs risk was evident only at middle (for low ACE) and high income (for low and high ACEs).

For Hispanic and NH Black immigrant households in the study, the broader societal benefits (e.g., reduced racial discrimination, safer living area) associated with moving up the income ladder may play a more significant role in buffering against ACEs. Although these households are disproportionately represented in substandard housing and face financial challenges, including difficulty affording health care [36, 37], they often demonstrate high levels of active and instrumental coping strategies [33, 34]. These coping strategies, along with the “healthy immigrant” phenomenon— where immigrants are a population group of highly educated, healthier, wealthier, motivated, and driven individuals relative to the general population in their home countries— may help buffer against the structural and economic stressors that increase risk of ACEs [38]. While this health advantage tends to diminish over time with longer U.S. residency, our study’s finding of a graded income effect may reflect differences in selective migration patterns and occupational opportunities that shape how income buffers against childhood adversity across immigrant subgroups [39, 40]. It is also important to note that NH Black immigrants exhibit high rates of selective migration and employment in occupational niches which facilitate social network development and informational support relevant for assimilation and reducing acculturation stress at any income level [41, 42].

However, assimilation in all aspects, notably language use, does not appear to be protective. Children in all three immigrant household groups with English as their primary language had higher odds of high ACEs (i.e., exposure to two or more ACEs); including low ACEs (exposure to one ACE) for NH White households. The use of household languages other than English among immigrant families may be indicative of protective cultural connections, positive bicultural identity, and family cohesion which is found to reduce likelihood of ACEs [13, 43, 44]. Theories of acculturation, including Berry’s Acculturative Stress Model [45] and Segmented Assimilation Theory [46, 47], suggest that immigrants who adopt the dominant culture—such as through exclusive use of the mainstream language (i.e., English)—may experience greater psychological and social stressors when cultural retention and familial cohesion are weakened [48]. Hence, language use may serve as an indicator of acculturation into the U.S. context, where greater assimilation—particularly at the expense of cultural and familial protective factors—could increase vulnerability to childhood adversity among immigrant families. Language loss or a shift to English-only particularly in households from non-English speaking countries can disrupt parent–child communication, reduce intergenerational support, and erode protective cultural identities, all of which are linked to increased vulnerability to stress and adversity [13, 48].

Across all three groups, having children in excellent/very good health was associated with reduced odds of high ACEs exposure; highlighting the far-reaching import of implementing policies that protect and prioritize the health, well-being, and safety of children in immigrant households [13]. Notably, insurance status, an important component of health maintenance, appears to have a different impact on ACEs exposure among the three groups. Compared to those with private insurance, children in NH White immigrant households with only public insurance or a mix of public and private insurance had a higher relative risk of experiencing high ACEs. In contrast, among children in NH Black households, only those with public insurance had increased relative risk of high ACEs. For Hispanic immigrant households, being uninsured and having both public and private insurance was linked to higher relative risk of high ACEs. This finding highlights that despite insurance access, children in these households may face other stressors—such as higher financial strain—that are associated with insurance eligibility and increase ACEs risk [49]. It also demonstrates the need for targeted health insurance literacy in addition to providing health insurance access.

The association of participation in other public benefit programs (WIC, TANF, or SNAP) with ACEs also varied by racial and ethnic group. Children in Hispanic immigrant households who received public benefits, had higher relative risk of exposure to both low and high ACEs. NH White immigrant children had higher relative risk of high ACEs (relative to no ACEs) if their households received public benefits. The exact reason for this is unclear, but it should be noted that this study’s data spanned the period of the “public charge rule,” which led many immigrant families to avoid public benefits for fear of jeopardizing their immigration status. The complex interplay between socio-cultural context, healthcare access, and immigration-related experiences and stressors in shaping ACEs among children in immigrant households [11, 50] is difficult to fully elucidate with cross-sectional survey data and deserves additional inquiry with more targeted datasets and qualitative insights in future work.

These findings should be interpreted in light of the study’s limitations. Although data used was nationally representative, it was cross-sectional, and causal inferences cannot be drawn. Additionally, the use of self-reported data on health and behaviors may introduce recall and social desirability bias. Defining children in immigrant households as those with at least one foreign-born parent does not consider whether the other parent is a non-immigrant, which could affect various determinants such as insurance and public benefits. Further, participants may not have fully understood the racial and ethnic categories used in the U.S., potentially leading to misclassification. In addition, the broad categories, such as “Hispanic” and NH “Black” immigrant, does not reflect the diverse nativities (e.g., Mexican, African, Caribbean) of the households grouped together which could have implications for health risks. The NSCH does not collect information on location of birth for foreign born respondents, so it is impossible to further disaggregate racial/ethnic groups by country or region of origin. Acculturation may also vary greatly between respondents and be associated with our outcomes, but we are not able to account for this beyond the inclusion of a variable for primary language in the household. Duration of residence in the United States, a potentially robust measure of acculturation was not included due to concerns around missing data. Although the NSCH survey is also administered in Spanish, language barriers may hinder accurate comprehension of the survey, including the ACEs measures. Moreover, these households might be less likely to report ACEs (e.g., maltreatment) due to cultural norms and fear of legal repercussions, which could bias the reported prevalence of ACEs. Additionally, the 9-item ACEs measure implemented in the NSCH does not assess the frequency, intensity, or chronicity of these ACEs [51]. Although the NSCH ACEs measure includes broader structural and environmental stressors such as neighborhood violence and discrimination—that may disproportionately affect immigrant populations— it did not account for immigration-specific stressors such as family separation due to migration or deportation, experiences of xenophobia, or fear related to immigration enforcement policies which are particularly salient for immigrant families but remain unmeasured. Therefore, it is possible that the ACEs measure may underestimate the true burden of childhood adversity in immigrant households.

## Conclusion

This study examined the structural and sociodemographic correlates of ACEs among children in immigrant households by race and ethnicity. Patterns of ACEs prevalence mirror those in the general population, with children in NH Black immigrant households at the highest risk. Within-group examination revealed common protective effects of economic and health stability against ACEs among the three immigrant racial and ethnic groups. The study’s within-group analysis reveals that risk and protective factors do not operate uniformly across racialized immigrant identities, even when controlling for socioeconomic status—an insight that is often obscured in aggregate or interaction-only models. These findings contribute to increasing evidence supporting the importance of disaggregating immigrant experiences and recognizing how intersecting social positions influence the conditions under which immigrant children are most at risk for adversity.

Acculturation-related stressors were also identified as a common risk determinant. However, significant differences emerged: income had a more graded protective effect for NH Black and Hispanic immigrant children, and household receipt of public benefits and insurance types showed varying associations among the groups. Study findings challenge assumptions that upward mobility and cultural adaptation uniformly benefit immigrant households, highlighting instead the complex and sometimes contradictory effects of the acculturation process (specifically, language assimilation) on family functioning and child well-being. These findings highlight the importance of applying an intersectional lens in broadening our current understanding of how structural and sociocultural factors interact with race, ethnicity, nativity, economic, and health system access to shape distinct patterns of risk and resilience across immigrant subgroups. It also emphasizes the need for tailored interventions and policies to address the unique challenges faced by immigrant families.

## Figures and Tables

**Fig. 3:**
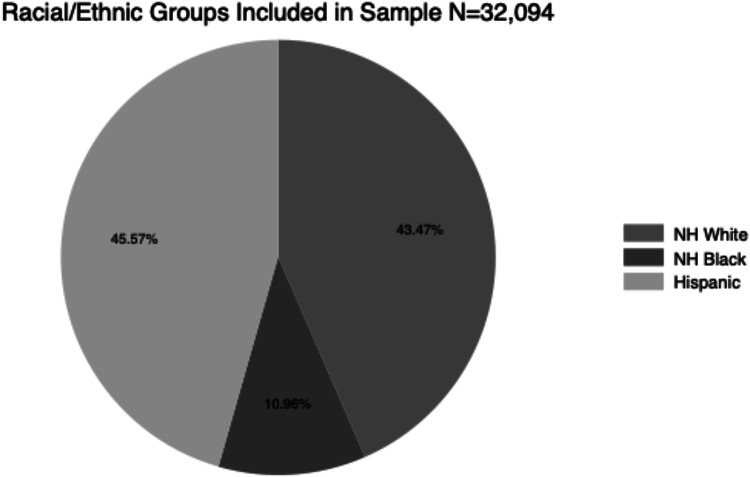
Racial/ethnic distribution of study sample (N=32,094)

## Data Availability

Data utilized for this study are publicly available via the United States Census Bureau and can be downloaded at this link https://www.census.gov/programs-surveys/nsch/data/datasets.html.

## Appendix A

See Table 3

**Table 3:** A. Descriptives of dropped observations

Variables	Unweighted N	Weighted Percentage	95% CI	
*Sample Child Race*				
NH White	1,019	15.2%	(0.13,	0.17)
NH Black	486	17.8%	(0.15,	0.21)
Hispanic	1,774	66.9%	(0.64,	0.70)
*Sample Child Biological Sex*				
Female	1,616	49.3%	(0.46,	0.53)
Male	1,663	50.7%	(0.47,	0.54)
*Sample Child Age*				
0–5 years	897	24.8%	(0.22,	0.28)
6–11 years	993	36.5%	(0.33,	0.40)
12–17 years	1,389	38.7%	(0.35,	0.42)
*Child is in Excellent or Very Good Health*				
Yes	2,784	85.2%	(0.82	0.88)
No	409	14.8%%	(0.12,	0.18)
*Household highest level of education*				
< 12 years	452	31.2%	(0.27,	0.35)
High school graduate	580	22.7%	(0.20,	0.26)
Some College or More	2,089	46.2%	(0.43,	0.50)
*Household Language*				
English	1,507	60.0%	(0.56,	0.63)
Other	1,349	40.0%	(0.37,	0.44)
*Child Insurance Type*				
Public only	913	48.7%	(0.44,	0.53)
Private only	1,239	30.9%	(0.28,	0.34)
Public and private	140	6.6%	(0.05,	0.09)
Uninsured	213	13.9%	(0.11,	0.17)
*Household has received public benefits in the last year*				
Yes	749	40.1%	(0.56,	0.64)
No	1,919	59.9%	(0.36,	0.44)
*Federal poverty level* ^a^				
Low income	859	34.2%	(0.30,	0.38)
Nearly poor	832	29.3%	(0.26,	0.33)
Middle income	840	22.4%	(0.19,	0.26)
High income	748	14.1%	(0.12,	0.16)

^a^Weighted percentage calculated using mi commands in STATA to account for multiple imputation.

N Varies due to missing data within sample.

## Appendix B

See Fig. 3

## Appendix C

See Table 4

**Table 4:** Expanded four-category Distribution of Total Adverse Childhood Experiences (ACEs) (N = 32,094)

ACE category	Unweighted N	Weighted Percentage	95% CI	
0	22,412	66.1%	(0.65,	0.67)
1	6,465	23.0%	(0.22,	0.24)
2	1,973	6.4%	(0.06,	0.07)
3	731	2.6%	(0.02,	0.03)
